# Detrimental effect of zwitterionic buffers on lysosomal homeostasis in cell lines and iPSC-derived neurons

**DOI:** 10.12688/amrcopenres.12903.1

**Published:** 2020-05-18

**Authors:** Sophie R. Cook, Rafael A. Badell-Grau, Emily D. Kirkham, Kimberley M. Jones, Brendan P. Kelly, Jincy Winston, Helen Waller-Evans, Nicholas D. Allen, Emyr Lloyd-Evans

**Affiliations:** 1 School of Biosciences, Sir Martin Evans Building, Cardiff University, Cardiff, CF10 3AX, UK; 2 Medicines Discovery Institute, Main Building, Cardiff University, Cardiff, CF10 3AT, UK

**Keywords:** Ca2+, HEPES, iPSC, lysosomal disease, lysosome, neuron, zwitterionic buffer

## Abstract

Good’s buffers are commonly used for cell culture and, although developed to have minimal to no biological impact, they cause alterations in cellular processes such as autophagy and lysosomal enzyme activity. Using Chinese hamster ovary cells and induced pluripotent stem cell-derived neurons, this study explores the effect of zwitterionic buffers, specifically HEPES, on lysosomal volume and Ca^2+^ levels. Certain zwitterionic buffers lead to lysosomal expansion and reduced lysosomal Ca^2+^. Care should be taken when selecting buffers for growth media to avoid detrimental impacts on lysosomal function.

## Abbreviations

ADF: advanced DMEM/F12 medium; DMEM: Dulbecco’s modified Eagle’s medium; HEPES: 4-(2-hydroxylethyl)-1-piperazineethanesulfonic acid; MES: 2-(N-Morpholino)ethanesulfonic acid; MOPS: 3-(N-Morpholino)propanesulfonic acid; NPC: Niemann-Pick disease type C; NPCs: iPSC-derived neural progenitor cell; PIPES: 1,4-piperazinediethanesulfonic acid; PPB: potassium phosphate buffer; Tris: 2-amino-2-(hydroxymethyl)-1,3-propanediol.

## Introduction

Good’s buffers, including 4-(2-hydroxylethyl)-1-piperazineetha-nesulfonic acid (HEPES), are commonly used zwitterionic buffers in cell culture^1^–^3^. These buffers were developed to be stable, membrane impermeant, and inert in biological reactions^2^, leading to their widespread use. Reports have, however, described zwitterionic buffers affecting biological processes; they induce morphological artefacts in fixed *Drosophila* tissue^4^, and alterations to autophagy and lysosomal enzyme activity in cultured cells^1^.

Lysosomes are acidic organelles, known as the recycling centre of the cell, since they breakdown cellular material. They also have important roles in cellular processes, including plasma membrane repair and cellular signalling as the second largest intracellular Ca^2+^ store^5^–^7^. Lysosomal dysfunction is a component of multiple diseases including Alzheimer’s, Parkinson’s and ~70 inherited lysosomal storage diseases^7^. Considering the reported impact of HEPES on lysosomal enzymes^1^, it is important to understand its effects, as well as other zwitterionic buffers, on lysosomal functions.

This study describes the effect of HEPES on lysosomal morphology and Ca^2+^ levels in control cells and those null for the lysosomal protein NPC1, whose function is lost in the lysosomal storage disease Niemann-Pick Type C (NPC). The findings highlight the importance of understanding the impact of growth media components on lysosomal functions.

## Methods

### Cells

Chinese hamster ovary (CHO) control H1 and NPC1-null M12 cells^8^ were grown as monolayers at 37°C/5% CO_2_ in Dulbecco’s Modified Eagle’s Medium (DMEM)/F-12 (Thermofisher) with 1% L-glutamine (Lonza), 10% heat-inactivated foetal bovine serum (Sigma/Pan Biotech) either with or without HEPES/other zwitterionic buffer at pH 7.4 (Thermofisher/Lonza).

Control induced pluripotent stem cell (iPSC)-derived neural progenitor cells (NPCs) were cultured on vitronectin-coated 6-well plates with E8 flex medium (Life Technologies) at 37 °C/5% CO_2_. Neural induction proceeded according to previous methods^9^ with modifications. Briefly, NPCs were derived in Advanced DMEM/F-12 (ADF) with GlutaMAX, penicillin/streptomycin (Life Technologies), 2% NeuroBrew 21 without retinoic acid (Miltenyi), LDN193189 (1 µM, Stemgent), SB431542 (10 µM, Abcam) and IWR1 (1.5 µM, Tocris). NPCs were expanded in ADF with 2% NeuroBrew 21 with retinoic acid (Miltenyi Biotec) and 10 ng/mL basic fibroblast growth factor. NPCs were terminally differentiated in SynaptoJuiceA (HEPES-free) for 7-days, followed by two weeks in SynaptoJuiceB (5.5 mM HEPES) according to^9^,^10^. Neurons were maintained in SynaptoJuiceB, both with and without additional 10 mM HEPES for 7 days.

### Buffers

All buffers (MOPS, PIPES, MES, PPB) were purchased from Sigma-Aldrich apart from HEPES (Thermofisher/Lonza) and Tris (Roche). With the exception of HEPES, which was purchased as a pre-made 1 M solution (pH 7.4), all buffers were made as 1 M stock solutions in mqH_2_O (or 1 M NaOH in mqH_2_O for PIPES), adjusted to pH 7.4 and filter sterilised through a 0.22 μm filter. PPB was adjusted to pH 7.4 by combining 1 M solutions of monobasic dihydrogen phosphate and dibasic monohydrogen phosphate. Buffers were added to culture media to a final concentration of 10 mM unless otherwise stated.

### Lysosomal measurements

Lysosomes were visualised in live cells in chamberslides (Ibidi) using 300 nM LysoTracker red or green (Life Technologies) in Dulbecco’s modified phosphate buffered saline (DPBS) for 15-minutes at room temperature, washed tree times with DPBS, and imaged using a Zeiss Axio Observer inverted microscope with Colibri LED light source and Zeiss Mrm CCD camera with Axiovision 4.8 software. Lysosomal area per cell was measured from LysoTracker fluorescence images in ImageJ 1.50i and 1.52n^11^ using the analyse particles function. LysoTracker fluorescence was measured in cells grown in Corning CellBIND 96-well plates (0.8x10^5 cells/well) using a SpectraMax® Gemini microplate reader (Molecular Devices).

Ca^2+^ measurements were done as described^12^ but with minor modifications for neurons, which were loaded with 1 µM Fura-2, AM (Stratech) without Pluronic F-127. Cells were imaged in Hank’s balanced salt solution (HBSS; 1 mM HEPES pH7.4, 10 µM CaCl_2_ and 1 mM MgCl_2_) using a Zeiss Axiovert 35 microscope with Cairn Optospin filter exchanger, Orca Flash 4.0 sCMOS camera and MetaFluor 7.10 software. For all experiments, ionomycin (Merck, 2 μM) was added to clamp intracellular Ca^2+^ stores followed by 500 µM Gly-Phe-β-naphthylamide (GPN, Abcam) to release lysosomal Ca^2+12^.

### Statistical analysis

All statistical analyses were performed in GraphPad Prism 8 software with data analysed by two-way ANOVA with Tukey’s post-hoc test or unpaired t-test as appropriate and where indicated in the figure legends.

## Results

**Figure 1.**
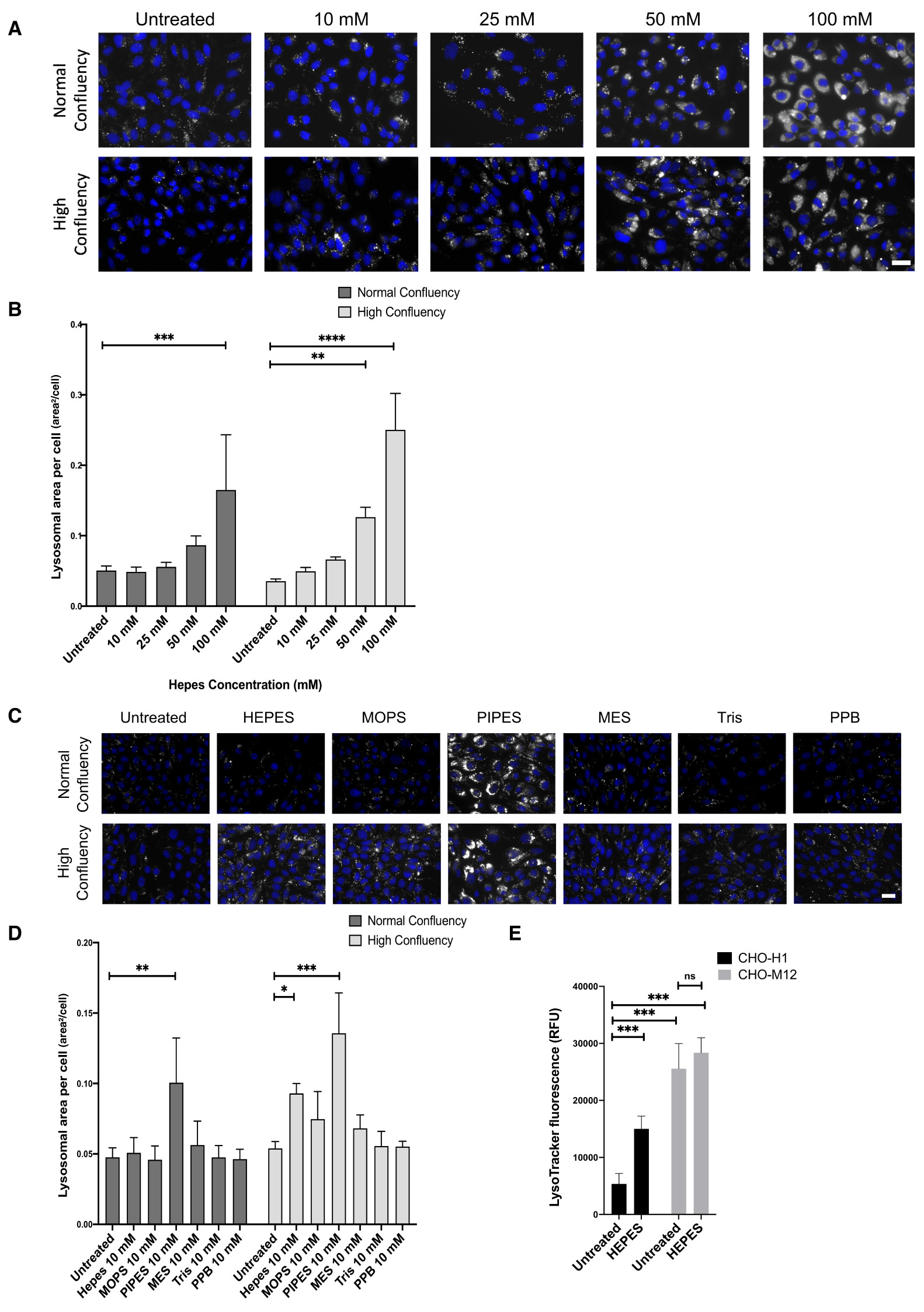
Changes in lysosomal area in cells grown in zwitterionic buffered media. (**A**) Representative images of control CHO-H1 cells loaded with LysoTracker green following 3-day treatment with the indicated concentrations of HEPES buffer. (**B**) Quantitative analysis of LysoTracker fluorescence from (**A**) as lysosomal area per cell, N=3–4 (9 cells analysed per repeat). (**C**) Representative images of CHO-H1 cells loaded with LysoTracker green following treatment for 3-days with 10 mM of the indicated buffers. PPB is potassium phosphate buffer. (**D**) Quantitative analysis of LysoTracker fluorescence from (**C**) as lysosomal area per cell, N=3–4 (8–9 cells analysed per repeat). (**E**) Fluorescence plate assay of control CHO-H1 cells and NPC1-null CHO-M12 cells loaded with LysoTracker green following 12-month growth in HEPES buffered medium, N=8. (**A**) and (**C**) Scale bars = 10 µm. (*p<0.05, ***p<0.001, ****p<0.0001, two-way Anova tests, post hoc Tukey's).

In agreement with previous findings of lysosomal enzyme dysfunction^1^, we observed HEPES-mediated lysosomal dysfunction in control CHO-H1 cells that was exacerbated at high cell confluency. Namely, a concentration-dependent expansion of the lysosomal system following 3-days growth in HEPES-containing buffer observed using LysoTracker (Figure 1A & B). Having confirmed this effect, we determined whether other zwitterionic buffers triggered similar effects. At a buffer concentration commonly found in growth media (10 mM), only PIPES, out of the six buffers tested, increased lysosomal area in control CHO-H1 cells over the 3-day treatment, that was also exacerbated by high cell confluency (Figure 1C & D).

To determine the long-term effects of growth in HEPES-containing media (10 mM), control CHO-H1 and the NPC1-null CHO-M12 cells were grown in this media for 12-months. When grown in HEPES-free media, there is a 4.8-fold increase in LysoTracker florescence, measured using a plate reader, in the lysosomal disease CHO-M12 cells, compared to control CHO-H1. Following 12 months of growth in media with HEPES, we observed no further increase in LysoTracker staining in NPC1-null CHO-M12 cells, whereas we observed a 2.8-fold increase in LysoTracker fluorescence in control CHO-H1 compared with control cells grown in HEPES-free media (Figure 1E). This illustrates that growth in HEPES-supplemented media impacts upon healthy lysosomal function and reduces the difference between control and lysosomal disease cells. This observation may have particular importance for cells requiring long-term growth in buffered media (e.g., iPSC-neurons).

**Figure 2.**
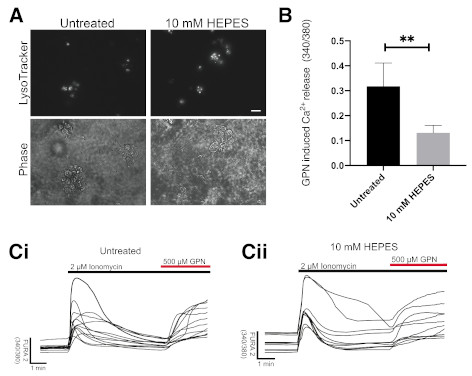
Growth of iPSC-derived neurons in HEPES containing media results in altered lysosomal Ca ^2+^ and causes lysosomal expansion. (**A**) Representative images of iPSC-derived neurons treated for 7 days in media containing 10 mM HEPES. Phase contrast microscopy images show location of neuronal cell bodies. Scale bar = 10 µm, N=3. (**B**) Following 7-day treatment in HEPES, lysosomal Ca^2+^ release, triggered by addition of 500 µM GPN, to induce osmotic lysis, after ionomycin to clamp other intracellular Ca^2+^ stores, was measured in iPSC-derived neurons, N=4 (7–14 cells analysed per repeat). (**C**) **i** and **ii** are Representative traces of Ca^2+^ release quantified in (**B**). (*p<0.05, unpaired t-test).

Therefore, we tested the effect of HEPES supplementation of SynaptoJuiceB on iPSC-neurons in culture for 7 days. Again, we observed an expansion of the lysosomal system (Figure 2A). Because zwitterionic buffers may act as a “proton sponge”, affecting both the volume and ion balance of lysosomes^13^, particularly lysosomal Ca^2+^ content which is dependent on lysosomal acidification^14^, we measured lysosomal Ca^2+^ content in these neurons. We observed significantly reduced lysosomal Ca^2+^ (2.2-fold) in neurons grown in the presence of 10 mM HEPES for 7 days compared to those grown without HEPES (Figure 2B). Raw data underlying this study are available at Figshare^15^.

## Discussion and conclusions

Our findings indicate that lysosomal expansion occurs after both short- and long-term culture in HEPES-buffered media and is exacerbated at higher cellular confluency. Moreover, this expansion impacts lysosomal function, namely lysosomal ion signalling in the form of reduced lysosomal Ca^2+^ content and is consistent with previous report of altered lysosomal glucosylceramidase activity in cells grown in HEPES^1^. Together, these data suggest that HEPES operates as a lysosomal proton sponge^13^,^16^. These observations provide a significant note of caution for lysosomal researchers, potentially impacting on lysosomal biochemical experiments such as measurement of pH^17^ or lysosomal purification methods^18^. Not all zwitterionic buffers have the same effects, only PIPES was also detrimental to lysosomal function, suggesting other zwitterionic buffers may be appropriate HEPES substitutes. Regardless, stringent consideration must be spent on buffer selection for relevant lysosomal studies.

## Data availability

### Underlying data

Figshare: Detrimental effect of zwitterionic buffers on lysosomal homeostasis in cell lines and iPSC-derived neurons. https://doi.org/10.6084/m9.figshare.12218441.v1^15^.

This project contains the following underlying data:

- Figure 1b HEPES concentration effect on lysosomal area (CSV). (Effect of different HEPES concentrations on lysosomal area.)
- Figure 1d Effect of zwitterionic buffers on lysosomal area (CSV). (Effect of each zwitterionic buffer on lysosomal area.)
- Figure 1e Effect of long term HEPES growth on LysoTracker fluorescence (CSV). (Fluorescence levels in CHO-H1 and NPC1-null CHO-M12 cells grown in HEPES for 12 months.)
- HEPES Effect on iPSC neurons Fura 2 GPN Ca^2+^ peak height data fig2b (CSV). (Effect of 7-day HEPES incubation on Ca^2+^ release in iPSC-derived neurons.)
- Untreated Iono GPN Fura 2 trace raw data fig2ci (CSV). (Raw Ca^2+^ release quantified from the above experiment, no HEPES.)
- 10 mM Hepes Iono GPN Fura 2 trace raw data fig2cii. (Raw Ca^2+^ release quantified from the above experiment, 10 mM HEPES.)
- Raw microscopy images (28 images; TIF).

Data are available under the terms of the Creative Commons Attribution 4.0 International license (CC-BY 4.0).
