# Supplementary material for: Detrimental effect of zwitterionic buffers on lysosomal homeostasis in cell lines and iPSC-derived neurons
Source: AMRC Open Res. Author manuscript; Available in PMC 2020 Jun 2. (PMC7266671; doi:10.12688/amrcopenres.12903.1)
Supplement: Peer review history

# Peer review: Reviewer response for version 1

**DOI:** 10.21956/amrcopenres.13973.r26491

**Published:** 2020-05-29

**Authors:** Marco van Eijk, Johannes Aerts

**Affiliations:** 1 Leiden Institute of Chemistry, University of Leiden, Leiden, The Netherlands; 2 Department Medical Biochemistry, Leiden Institute of Chemistry, Leiden, The Netherlands

Together with a colleague the paper by Cook et al has been reviewed. The paper is clearly written and scientifically sound. We feel the paper nicely builds on the earlier work of Tol and colleagues (Autophagy 2018)^1^, who demonstrated HEPES triggered lysosomal biogenesis. The work presented here is relevant due to the tested cell types and it adds to the understanding how HEPES impacts lysosomes, now also revealing a Ca2+ response.

 Some minor remarks, but the manuscript is suitable for passing peer review.

 The minor questions open are:

- The commercial buffers normally are composed of 25mM HEPES. In the first figure 50 and 100mM have been used. Did authors verify toxicity?
- Have authors an idea if the MiT-TF family is involved in the studied cell types (small statement in discussion will do).

Is the work clearly and accurately presented and does it cite the current literature?

Yes

If applicable, is the statistical analysis and its interpretation appropriate?

Yes

Are all the source data underlying the results available to ensure full reproducibility?

Yes

Is the study design appropriate and is the work technically sound?

Yes

Are the conclusions drawn adequately supported by the results?

Yes

Are sufficient details of methods and analysis provided to allow replication by others?

Yes

Reviewer Expertise:

Lysosomal storage disorders, obesity, metabolic inflammation.

We confirm that we have read this submission and believe that we have an appropriate level of expertise to confirm that it is of an acceptable scientific standard.

## References

[1] TolMJ: HEPES activates a MiT/TFE-dependent lysosomal-autophagic gene network in cultured cells: A call for caution. *Autophagy*.2018;14(3) : 437-449 10.1080/15548627.2017.1419118 29455584PMC5915011

# Peer review: Reviewer response for version 1

**DOI:** 10.21956/amrcopenres.13973.r26492

**Published:** 2020-05-21

**Authors:** Stephane Lefrancois

**Affiliations:** 1 Centre INRS-Institut Armand-Frappier, National Institute of Scientific Research, Laval, QC, Canada

The manuscript by Cook at al aims to explore the effect of zwitterionic buffers on lysosome function and morphology. 

 This work is of importance to people in the field of lysosomal biology and other cell biologists performing experiments where the morphology and function of lysosomes are being considered.

 They confirmed the effects of HEPES of lysosomal function and they tested other buffers, finding an effect only with PIPES.

 The results are well presented, properly quantified with statistical analysis. As the use of iPS cells is becoming more prominent in research, they extended their observation beyond CHO cells.

 They show the negative effects of HEPES buffer often used in cell culture media. 

 These results should be considered by all biologists when culturing cells and how HEPES and other reagents could affect data.

Is the work clearly and accurately presented and does it cite the current literature?

Yes

If applicable, is the statistical analysis and its interpretation appropriate?

Yes

Are all the source data underlying the results available to ensure full reproducibility?

No source data required

Is the study design appropriate and is the work technically sound?

Yes

Are the conclusions drawn adequately supported by the results?

Yes

Are sufficient details of methods and analysis provided to allow replication by others?

Yes

Reviewer Expertise:

Cell biology, lysosome biology

I confirm that I have read this submission and believe that I have an appropriate level of expertise to confirm that it is of an acceptable scientific standard.
